# Integrating Semantic Information into Multiple Kernels for Protein-Protein Interaction Extraction from Biomedical Literatures

**DOI:** 10.1371/journal.pone.0091898

**Published:** 2014-03-12

**Authors:** Lishuang Li, Panpan Zhang, Tianfu Zheng, Hongying Zhang, Zhenchao Jiang, Degen Huang

**Affiliations:** 1 School of Computer Science and Technology, Dalian University of Technology, Dalian, China; 2 Faculty of Chemical, Environmental and Biological Science and Technology, Dalian University of Technology, Dalian, China; 3 Department of Pathology, Dalian Medical University, Dalian, China; Huazhong University of Science and Technology, China

## Abstract

Protein-Protein Interaction (PPI) extraction is an important task in the biomedical information extraction. Presently, many machine learning methods for PPI extraction have achieved promising results. However, the performance is still not satisfactory. One reason is that the semantic resources were basically ignored. In this paper, we propose a multiple-kernel learning-based approach to extract PPIs, combining the feature-based kernel, tree kernel and semantic kernel. Particularly, we extend the shortest path-enclosed tree kernel (SPT) by a dynamic extended strategy to retrieve the richer syntactic information. Our semantic kernel calculates the protein-protein pair similarity and the context similarity based on two semantic resources: WordNet and Medical Subject Heading (MeSH). We evaluate our method with Support Vector Machine (SVM) and achieve an F-score of 69.40% and an AUC of 92.00%, which show that our method outperforms most of the state-of-the-art systems by integrating semantic information.

## Introduction

Extracting biomedical information from the literature is an important research topic in the field of biomedical natural language processing (BioNLP). With the rapidly growing number of research papers, it is becoming increasingly difficult for biomedical experts to detect the protein information manually. Thus automated protein-protein interactions extraction (PPIE) from biomedical literature has attracted substantial attention. PPIE is of great value on the application and practical significance, particularly establishing the network of protein knowledge, predicting the protein-protein relations and developing new drugs and so on.

At present, many methods have been used to extract protein-protein interaction relations. Most of these methods for PPI extraction task are pattern-based methods [1]–[3] and statistical machine learning methods [4]–[7].

The Pattern-based methods employ pre-defined patterns and rules to match the labeled sequence. Fundel et al. [1] developed RelEx system for PPI extraction, based on natural language pre-processing producing dependency parse trees and the application of a small number of simple rules to these trees. Huang et al. [2] used a dynamic programming algorithm to compute the distinguishing patterns by aligning relevant sentences and key verbs to describe protein interactions, achieving good results. Ono et al. [3] presented a method for extracting PPI by searching with protein names, word patterns and simple part-of-speech (POS) rules. However, the pattern-based method does not cover all of the modes and cannot produce new models.

The statistical machine learning methods can effectively avoid the disadvantages of the above methods. The current machine learning methods for PPI extraction task involve feature vectors based and kernel-based methods. Liu et al. [4] investigated the combination of diverse lexical, syntactic and particularly dependency information for feature-based protein-protein interaction extraction using SVM, achieving a promising F-score of 54.7% on the AIMED corpus. However, the feature-based methods cannot utilize the complex structure information in a sentence. Therefore researchers use kernels rather than a single feature vector. Airola et al. [5] proposed a graph kernel- based approach for the automated extraction of PPI from the scientific literature, achieving 56.4% F-score and 84.8% AUC on the AIMED corpus. Miwa et al. [6] proposed a method, which combined the kernels with several syntactic parsers. Their method combined the subset tree kernel and graph kernel, obtaining an F-score of 61.9% on the AIMED corpus. Yang et al. [7] presented a weighted multiple-kernel learning-based approach combining four kernels: feature-based, tree, graph and POS path, accomplishing a 64.41% F-score on the AIMED corpus.

However, few studies employed the semantic knowledge obtained from ontologies such as MeSH [8] and WordNet [9]. To make the most of the semantic resource, this paper proposes a semantic kernel, and combines it with the feature-based kernel and tree kernel to extract PPI. In the tree kernel, we extend SPT by a dynamic extended strategy to capture the richer syntactic information. In the semantic kernel, we combine the protein-protein pair similarity with the context similarity.

The remainder of the paper is organized as follows: The detailed implementation of our method is described in Section “Methods”. Section “Results” presents our experimental results and the comparisons with other systems. The discussion and error analysis are illustrated in Section “Discussions”, followed by Section “Conclusions and Future Work”.

## Methods

Our method is based on SVM and three distinctive types of kernels are combined, i.e., feature-based, tree and semantic kernel.

### Feature-based Kernel

The following features are used in our feature-based kernel.

#### Word feature

The word features are the most basic and important feature. There are four sets of words features in our method.

1. Words from protein names: all the words in two protein names are included.
2. Words between two protein names: these features include all words that are located between two protein names. If no word appears between two protein names, the feature will be “NULL”.
3. Words surrounding two protein names: these features include the left *n* words of the first protein name and the right *n* words of the second protein name. *n* is set to be five in our experiments. If there are no words surrounding two protein names, “NULL” will be used.
4. Interaction term: the interaction word (such as “regulate”, “interact”, “modulate”) often implies the existence of PPI. If only one keyword appears between or among the surrounding words of two protein names, the keyword is added into the interaction term feature. If there is more than one keyword, the first one will be used. If no keyword appears, the feature will be set to “NULL”.

#### Distance feature

From the corpus, we find that the shorter distance (the number of words) between the two proteins is, the more likely the protein pair has interaction relation. The distance feature can be divided into two classes.

1. The number of the non-proteins between two proteins (Word-Num)

If Word-Num≤3, the feature value will be set to “1”; if 3<Word-Num≤6, the value will be set to “2”; if 6<Word-Num≤9, it will be set to “3”; else, it will be set to “4”.

2. The number of the proteins between two proteins

If no other proteins appear between the two proteins, the feature value will be set to “0”; otherwise, it is the number of other proteins.

### Tree Kernel

**Figure 1:**
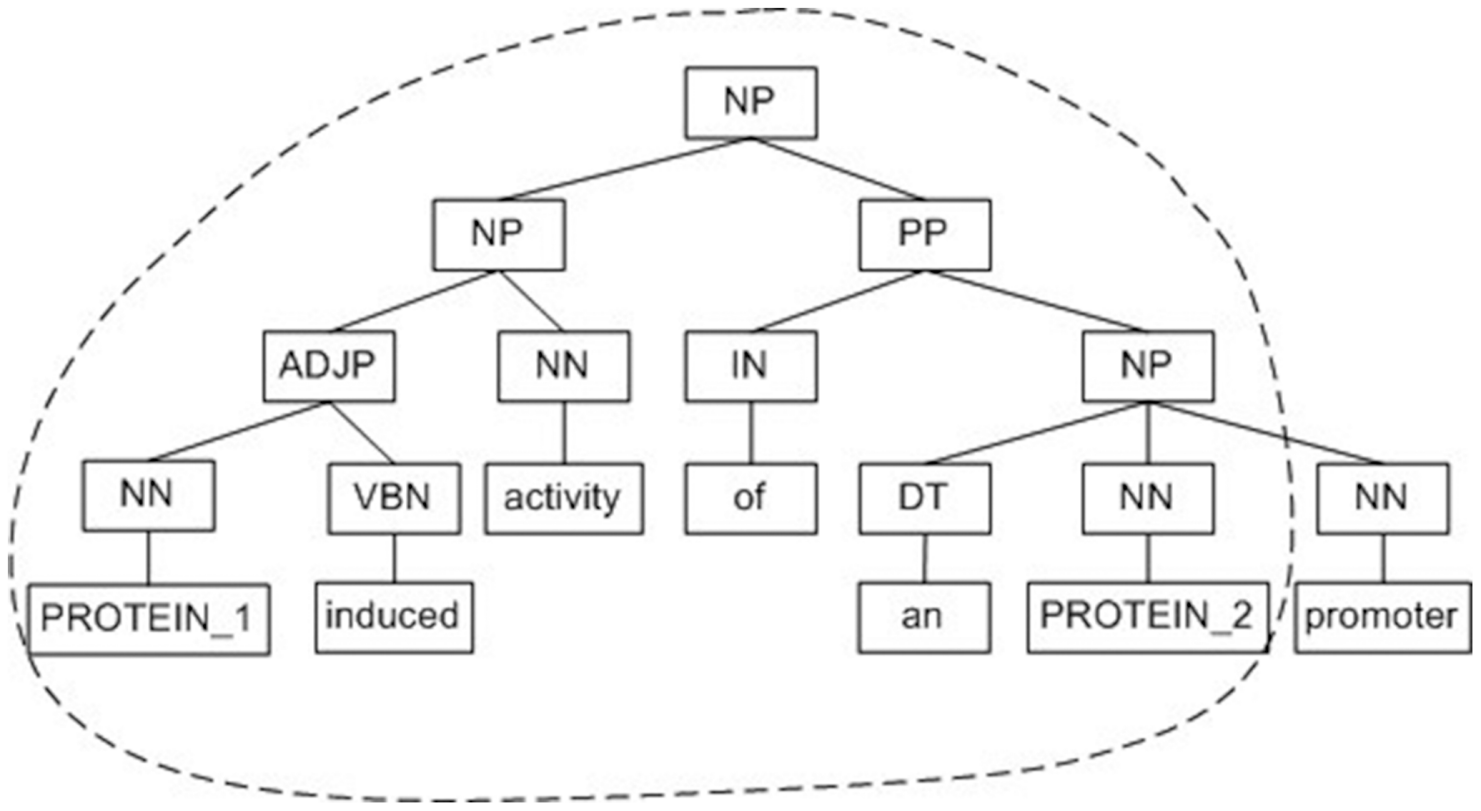
The MCT and the SPT of the sentence, “PROTEIN coexpression largely abolished *PROTEIN_1* induced activity of an *PROTEIN_2* promoter”. The SPT between the focused proteins is shown in the dotted line and MCT is the whole figure.

Our tree kernel adopts the convolution tree kernel proposed by Collins et al. [10]. A convolution tree kernel aims to capture the structured information in a sentence. It calculates the syntactic structure similarity between two parse trees by counting the number of common sub-trees [10], [11]. In order to focus on the most relevant information to relations, a standard tree kernel is defined on the Minimum Complete Tree (MCT). It is the sub-tree rooted by the nearest common parent node of the two proteins under consideration. In our tree kernel, the protein pairs in the sentence are replaced by PROTEIN_1 and PROTEIN_2, and the other protein names in the same sentence are replaced by PROTEIN. Stanford Parser [12] is used to parse the sentence. However, MCT includes too much left and right context information, which may elicit many noisy features. Zhang et al. [13] proposed five pruning strategies and found that the Shortest Path enclosed Tree (SPT) performed best. SPT is the smallest common sub-tree including the two proteins. In other words, the sub-tree is enclosed by the shortest path linking the two proteins in the parse tree. Figure 1 illustrates the different representation of MCT and SPT of a relation instance. The candidate interaction pair is marked as PROTEIN_1 and PROTEIN_2, the other proteins are marked as PROTEIN. The SPT between the focused proteins is shown in the dotted line and MCT is the whole figure.

**Figure 2:**
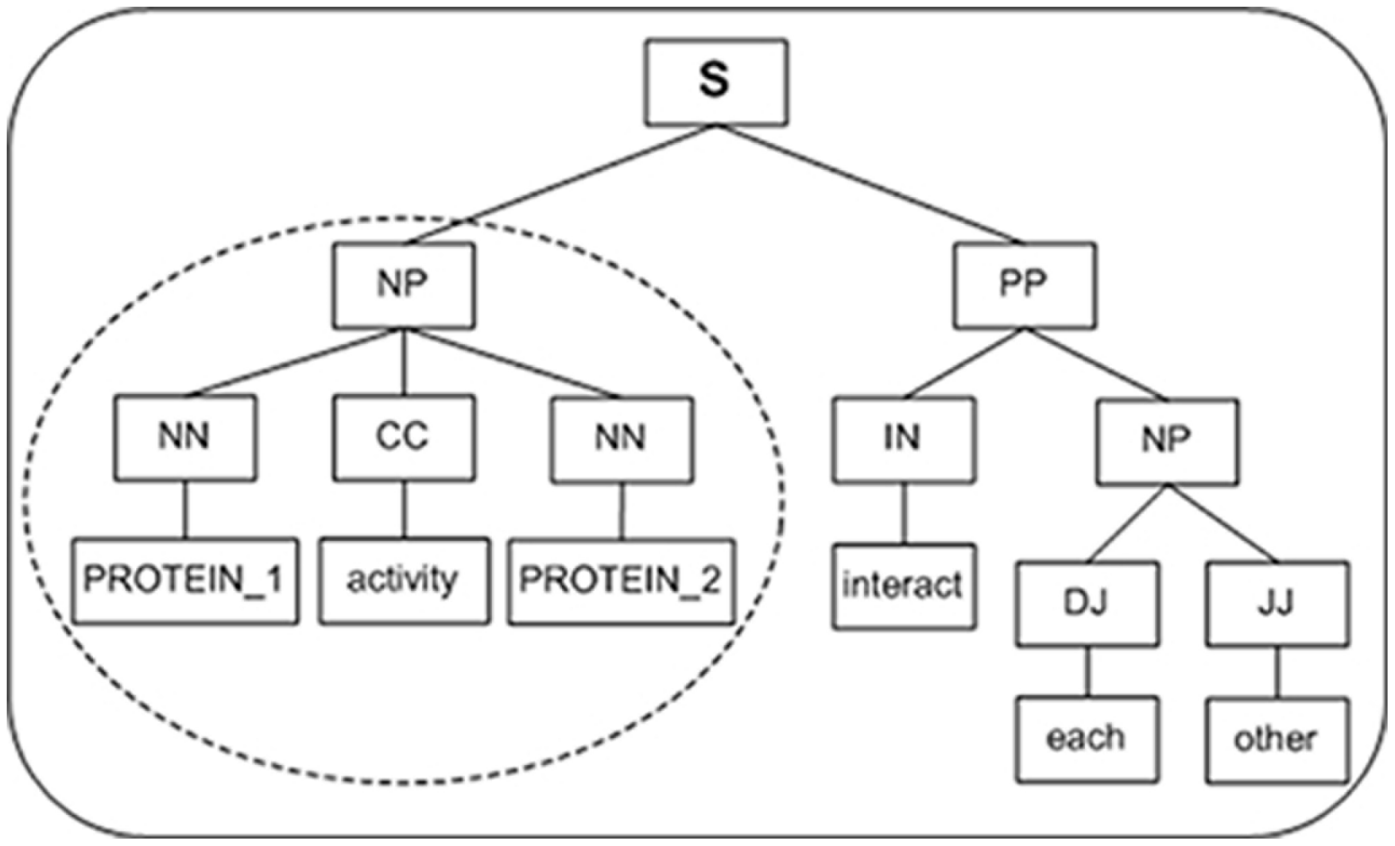
An example of the extension of SPT. The sentence is “PRITEIN_1 and PROTEIN_2 interact with each other”. The original SPT is in dotted circle and the extended SPT is in solid circle.

Despite the better performance of SPT, in some cases, the information contained in SPT is not sufficient to determine the relation between two proteins. For example, the word “interact” is critical to determine the relation between PROTEIN_1 and PROTEIN_2 in the sentence “PROTEIN_1 and PROTEIN_2 interact with each other”. However, it is not contained in the SPT (dotted circle in Figure 2). By analyzing the corpus, the quantity of nodes in these SPTs are found often less than seven, so they include less information except the two protein names. Here, we propose a novel dynamic extended strategy. By default, the tree kernel adopts the original SPT; When the number of the nodes in a SPT is smaller than 7:

1. If SPT is different with MCT, SPT will be replaced with MCT;
2. Otherwise, SPT will be replaced with the MCT rooted by the parent node of the root of the original SPT.

Thus the extended SPT (solid circle in Figure 2) will include richer context information than the original SPT. In the above example, SPT and MCT are the same, then the SPT will be extended from “*(NP (NNP PROTEIN_1) (CC and) (NNP PROTEIN_2))*” to “*(S (NP (NNP PROTEIN_1) (CC and) (NNP PROTEIN_2)) (VP (VBP* ***interact*** *) (PP (IN with) (NP (DT each) (JJ other)))))*”, which includes the keyword “*interact*” and richer context information. We call it Dynamic Extended Tree (DET).

### Semantic Kernel

Our semantic kernel consists of two parts: Protein Pair Similarity (*sim_pp_*) and Context Semantic Similarity (*sim_con_*). They are mainly based on the following two assumptions:

1. If the semantic information between two different protein pairs is closer, the instances including them are more likely to have the same type of relations;
2. If two instances have similar context, they will have the same type of relations.

#### Protein pair similarity

The semantic information of proteins can be derived from MeSH. MeSH is a taxonomic hierarchy of medical and biological terms suggested by the U.S National Library of Medicine. Hliaoutakis et al. [14] investigated several semantic similarity measures between proteins in MeSH and verified that the similarity measures outperformed others on MeSH as proposed by Li et al. [15] and Lin et al. [16]. Li et al.’s method was based on edge counting and Lin’s method was based on the information content of each concept. Therefore, we propose a hybrid way by combining them to calculate the similarity between two proteins as formula (1), where 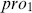 and 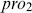 are two proteins in a sentence, 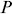 represents the protein pair.
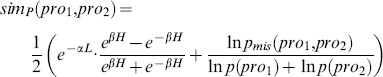
(1)


In formula (1), 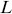 and 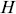 are the length of shortest path and the larger depth between 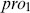 and 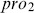 in MeSH database respectively, 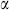 and 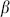 are parameters scaling the contribution of 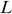 and 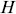, 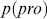 is the probability of encountering a protein 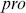, 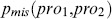 is the information content of the shared parents of two terms 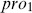 and 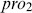.


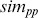
 is defined to calculate the similarity between two protein pairs 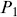 and 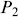 as formula (2):
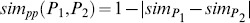
(2)


where 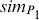 is the similarity between the two proteins in 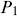, 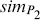 is the similarity of 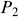.

#### Context semantic similarity

WordNet is a well-known upper ontology, storing rich semantic information. Unlike MeSH, entries in WordNet are more common. We take advantage of WordNet to measure the context semantic similarity. All words are treated as a context except protein names and stop words in a sentence.

**Figure 3:**
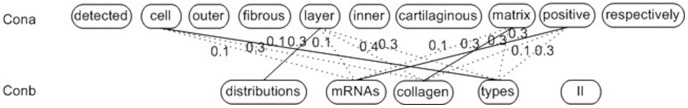
An example of the maximum matching in the bipartite graph. *Cona* and *Conb* are two different contexts. The maximal matching between *Cona* and *Conb* is flagged with solid lines. Edges with weight 0 are bypassed and all weights are rounding in this figure.

**Figure 4:**
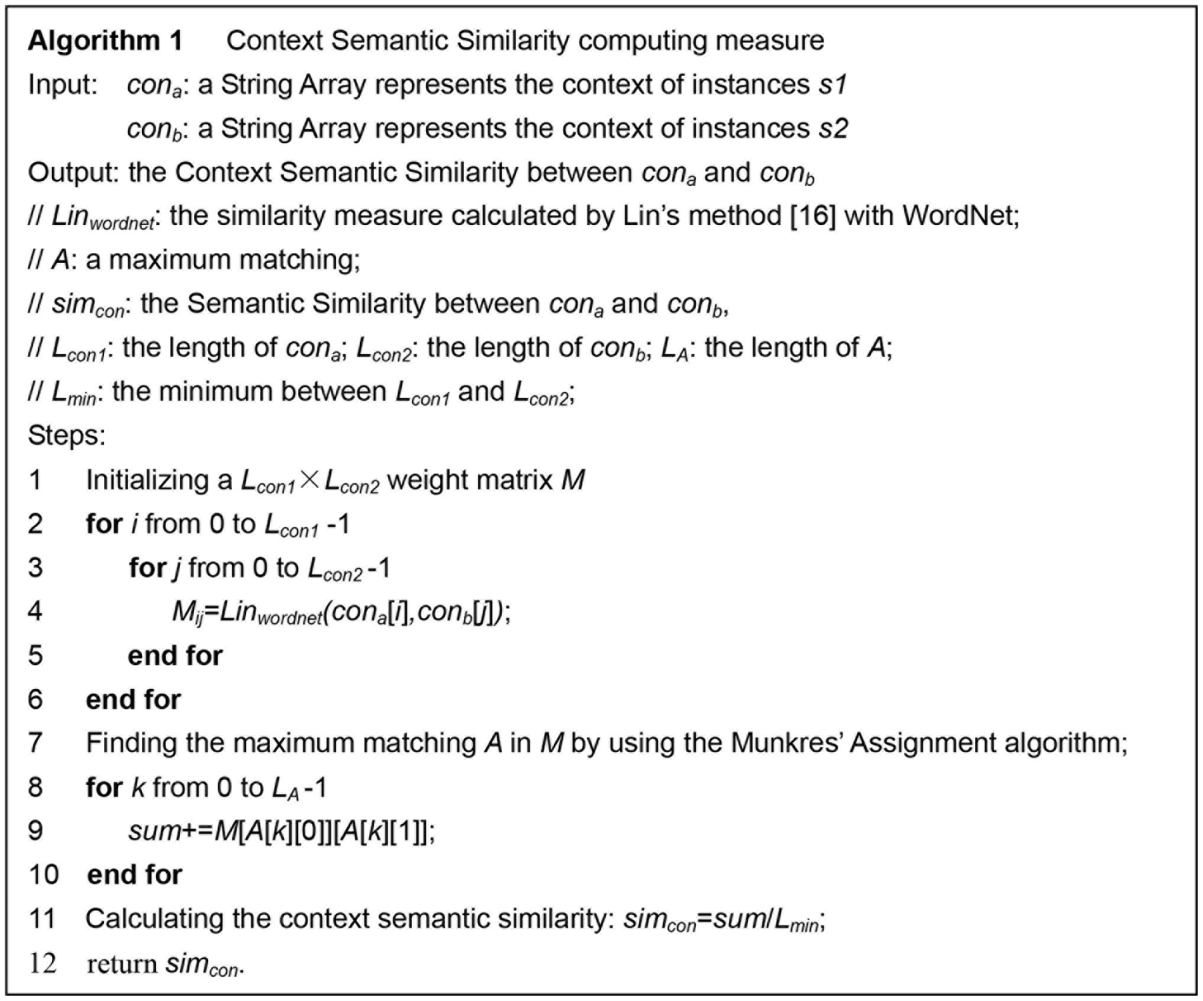
Context Semantic Similarity calculation based on Munkres’ Assignment Algorithm. Each element in the matrix *M* is calculated by Lin et al.’s method [16].


According to previous assumptions, the similar contexts indicate the same relations. We exploit the Kuhn-Munkres Algorithm [17] (also known as Munkres’ Assignment algorithm) to calculate the context semantic similarity. In our approach, two different contexts are regarded as two disjoint sets 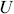 and 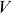 in a bipartite. The words in the contexts are vertices in 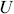 and 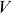 respectively. Lin’s method [16] is used to calculate the weight between a vertex in 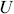 and a vertex in 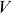. Therefore, a weighted completed bipartite graph can be modeled. The context semantic similarity can be obtained by finding the maximum matching in the bipartite graph. An example of maximum match is demonstrated in Figure 3. The algorithm to calculate the context semantic similarity based on Munkres’ Assignment Algorithm is exhibited in Figure 4.

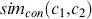
 is defined to calculate the Context Semantic Similarity as formula (3):
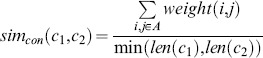
(3)


where 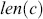 is the number of words in context 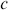, 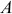 is the maximum matching of 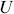 and 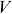 obtained by Munkres’ Assignment Algorithm, 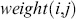 stands for the similarity between the 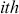 vertex in 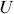 and the 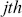 vertex in 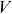.

#### Semantic kernel

The semantic kernel (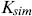) is defined as formula (4):
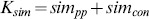
(4)


where 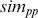 represents Protein Pair Similarity and 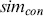 represents Context Semantic Similarity.

### Ensemble Kernel

Each kernel can capture some aspects of the available information in a sentence while losing other aspects. Combining different kernels produce a new useful similarity measure to reduce the danger of missing important information. We define the liner ensemble kernel as formula (5):
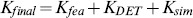
(5)


where 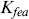 represents the feature-based kernel, 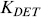 stands for the dynamic extended tree kernel, and 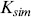 is the semantic kernel.

## Results

### Evaluation Measures

We evaluate our method on the AIMED corpus [18]. The corpus is a popular dataset for the evaluation of PPI extraction methods, consisting of 225 biomedical paper abstracts available in Medline.

There are many assessing methods for PPI extraction. Von Mering et al. [19] used accuracy to measure the performance of protein-protein interactions. Xia et al. [20] used the sensitivity, precision and accuracy for assessment. A ROC curve was drawn in You et al’s method [21]. A special versus sensitivity analysis was performed in [22]. In these discriminative assessing methods [19]–[22], the performance of protein-protein interaction can all be measured by the quantity of true positives (TP), true negatives (TN), false positives (FP), and false negatives (FN). In this study, the AUC measure (the area under the ROC [receiver operating characteristics] curve) and the balanced F-score are used for quantifying the performance of our methods, both of which can be calculated by TP, TN, FP and FN. F-score is the harmonic mean of P and R, where P denotes Precision and R denotes Recall. The ROC curve is a plot of the true positive rate (TPR) vs. the false positive rate (FPR) for different thresholds. AUC has the important property of being invariant to the class distribution of the used dataset and has been advocated for performance evaluation in the machine learning community [23]. Because of these two different points of view, the best result in AUC may be different from the best result in the F-score. Here, we report both results.

In the following experiments, our results are obtained with 10-fold cross-validation.

### Experimental Results

In this section, we firstly discuss the effectiveness of SPT and its dynamic extended tree (DET). Then the results of different kernels on AIMED corpus are presented.

#### Effectiveness of our DET kernel

Table 1 shows the performance of different tree kernels.

**Table 1:** Effectiveness of our DET kernel.

Kernel	P	R	F	AUC
Our SPT	73.74%	40.10%	51.95%	85.50%
Our DET kernel	75.01%	41.30%	53.27%	87.00%
Tree Kernel [7]	43.71%	64.65%	52.24%	79.19%

We achieve an F-score of 51.95% and an AUC of 85.50% with the original SPT. With the dynamic extended tree, the F-score and AUC are improved to 53.27% and 87.00% respectively (1.32% increase in F-score and 1.50% increase in AUC). Yang et al. [7] proposed a tree kernel consisting of SPT Extension and Dependency Extension, while our tree kernel adopted SPT Extension only. Our method outperforms theirs by 1.03% in F-score and 0.81% in AUC. Yang et al. [7] used a static extended strategy to extend SPT, namely, if the number of leaf nodes is smaller than four, the parent node of the root node of the original SPT will be used as the new root node. Our tree kernel (SPT extension) is a dynamic one, containing information most relevant to PPI extraction.

#### Effectiveness of different kernels

The performance of different kernels is shown in Table 2.

**Table 2:** Experimental results of different kernels.

Kernel	P	R	F	AUC
K_fea_	73.63%	37.50%	49.69%	88.70%
K_fea_+K_DET_	74.93%	55.40%	63.70%	90.80%
K_fea_+K_DET_+sim_con_	71.48%	65.10%	68.14%	90.70%
K_fea_+K_DET_+sim_pp_	72.52%	66.50%	69.38%	92.00%
K_fea_+K_DET_+K_sim_	72.45%	66.70%	69.46%	92.00%

The experimental results show that, better performance is achieved if two or more individual kernels are combined. When the tree kernel is combined with the feature-based kernel, the performance is improved by 14.01% in F-score and 2.10% in AUC. When further combined with context semantic similarity, the F-score is improved by 4.44% while AUC slightly reduces by 0.10%. Added the protein pair similarity, the F-score and AUC are improved to 69.38% and 92.00% respectively (5.68% increases in F-score and 1.20% in AUC). Finally, the addition of 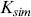 (that is, the context similarity is integrated to the protein pair similarity) increases 0.08% in F-score (69.46% vs. 69.38%), and AUC performs same with 92.00%.

From the above experimental results, we can see: (1) the context semantic similarity does not contribute more than the protein pair similarity (4.44% vs. 5.68% in F-score); (2) when the context semantic similarity is combined with the feature-based, DET and protein pair similarity, the F-score only increases 0.08% and AUC does not change. One reason is that all words in the sentence are treated as the context except for protein names and stop words, which might introduce random noise features to degrade the performance when combined with protein pair similarity; the other is that the sparseness of the training set affects the performance. No common or similar words exist in the corresponding contexts, resulting in the lower context semantic similarity. The same or similar contexts indicate the same relation, thus the sparseness of the training set produces certain effect on the performance. However, the kernels with context semantic similarity always perform better than the kernels without integrating any semantic information.

**Figure 5:**
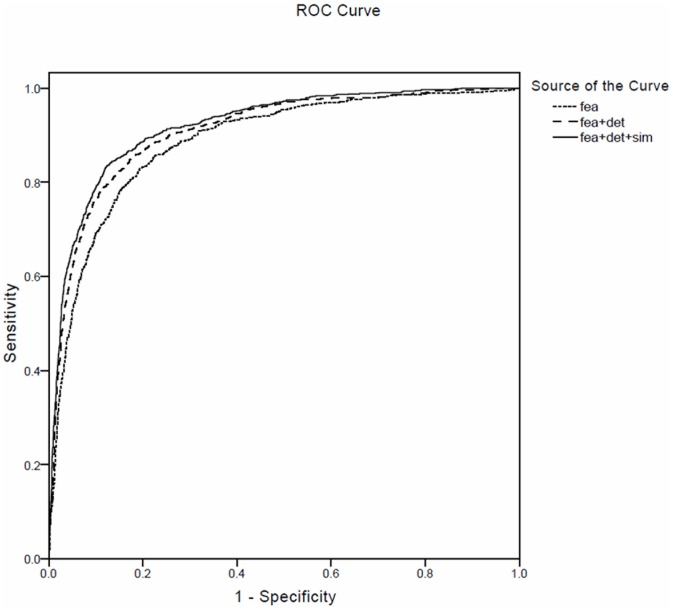
The ROC curves of three experiments: the feature-based kernel, the combination of feature-based kernel and DET kernel, and the kernel integrating three individual kernels.

Figure 5 depicts the ROC curves of different prediction results based on feature-based kernel, kernel with addition of DET kernel, and kernel integrating three individual kernels. It shows obviously that the combination of three kernels performs best on AUC.

### Comparisons

This section presents the comparisons between our method and some state-of-the-art works on the AIMED corpus.

Table 3 shows our approach achieves the best performance with an F-score of 69.46% and an AUC of 92.00%. The methods in [6], [7], [24] also adopted an ensemble kernel to extract PPI. Miwa et al. [6] combined the tree kernel with graph kernel and reached 61.90% in F-score and 87.60% in AUC. Yang et al. [7] combined several kernels to extract PPI: feature-based, tree, graph and POS path kernel. Our method outperforms it by 5.05% and 3.54% in F-score and AUC respectively. Miwa et al.’s methods [24] attained an F-score of 63.50% and an AUC of 89.70%. They integrated multiple layers of syntactic information for PPI extraction. Unlike the three above systems, Li et al. [25] combined the new features generated by feature coupling generalization (FCG) with local lexical features without any syntactic and semantic information, obtaining an F-score of 63.54% and an AUC of 87.24%. The performance of our method still outperforms it by 5.92% in F-score and 4.76% in AUC. Overall, our method achieves the best performance.

**Table 3:** Comparisons between our method and some state-of-the-art systems.

Methods	P	R	F	AUC
Miwa et al. [6]	58.70%	66.10%	61.90%	87.60%
Yang et al. [7]	57.72%	71.07%	64.41%	88.46%
Miwa et al. [24]	60.40%	69.30%	63.50%	87.90%
Li et al. [25]	60.47%	68.31%	63.54%	87.24%
Ours	72.45%	66.70%	69.46%	92.00%

## Discussions

### Discussions

We combine the dynamic extended tree kernel and semantic kernel with feature-based kernel to extract PPI. Experimental results show that our PPIE method outperforms most of the state-of-the-art systems. The effectiveness analysis is as follows.

1. Effective Individual Kernel

Firstly, a novel dynamic extended method is proposed. The extended SPT contains the information most relevant to PPI extraction. For example, the protein pair “HFE” and “TfR” in the instance S1 will be replaced by PROETIN_1 and PROTEIN_2 in our tree kernel. And the corresponding SPT and DET are also given as follows.

*S1*: “The ***HFE***-***TfR*** complex suggests a binding site for transferrin on TfR and sheds light upon the function of HFE in regulating iron homeostasis.”

*SPT: (NP (NN PROTEIN_1)(NN PROTEIN_2)*

*DET: (NP (DT The)(NN PROT_1)(NN PROT_2)(NN* ***complex*** *))*

The protein pair “HFE” and “TfR”, classified as a false negative case by SPT, can be classified correctly as a true positive one by DET, because DET includes the interaction word “complex” to get richer context information.

Secondly, the semantic kernel takes advantage of the semantic knowledge derived from MeSH and WordNet, to calculate the semantic similarity between two instances. Results in Table 2 show that, with addition of semantic information, the performance of our PPI extraction algorithm is improved. For example, the instance S2 contains the interaction term “interact”, but the feature-based kernel and DET kernel both classified the protein pair “JAK2” and “Raf-1” as a false negative instance. However, they can correctly be classified as a true positive one by introducing the semantic kernel. The reason is that the semantic resource provides richer and deeper information from the perspective of language understanding.

*S2*: “***JAK2***, a member of the Janus kinase superfamily was found to interact functionally with ***Raf-1***, a central component of the ras/mitogen-activated protein kinase signal transduction pathway.”

2. Combination of Kernels

We propose an ensemble kernel including the feature-based kernel, tree kernel and semantic kernel. The feature-based kernel is simple and efficient, but can not capture the syntactic and semantic information. The tree kernel can calculate the similarity between two shortest paths by utilizing the syntactic structure information. The semantic kernel considers the deep semantic information. Given their individual characteristics, different kernels calculate the similarity between two instances from different aspects. Thus, combining different kernels produce a new useful similarity measure to reduce the danger of missing important information.

From the above analysis, it can be seen that our PPI extraction algorithm based on integrated kernels is statistically effective.

### Error Analysis

Our method improves the performance of PPI extraction on AIMED, but there are still some disadvantages. A few error examples are listed here. In these instances, the protein pairs focused are marked with bold slash.

1. The feature-based kernel considers the negative words as an indicative feature. When a negative word exists in an instance, it tends to be classified as a negative one. However, it may lead to a false negative instance. For example, it can be observed that “not” exists in the instance “Moreover, mutation of ***Raf-1*** residues 143–145 impairs binding of 14-3-3, but **not** Ras, to the ***Raf-CRD***”. Our method classifies it as a negative instance while it is a positive one in fact.
2. Some instances have no sufficient information. For example, the protein pair in the instance “Sos and ***80K-H***, ***pp66***” will be classified as a false negative instance. The instances have no adequate lexical features, syntactic features and rich context information, except for the protein names.
3. Some errors in the corpus’s annotation. This case will lead to some incorrect classifications.
4. Confined by the complexity of the PPI expression as well as the quantity and quality of the corpus, some inevitable false classifications will be generated which are not itemized here.

## Conclusions and Future Work

In this paper, a multiple-kernel learning-based approach is presented for the protein-protein interaction extraction. The approach contains the following three individual kernels: feature-based kernel, tree kernel and semantic kernel. Our method aims to improve the performance of PPIE by embedding the semantic information into kernel calculation. Experiments and comparisons demonstrate that our PPIE method works better than the state-of-art systems on the AIMED corpus, with an F-score of 69.46% and an AUC of 92.00%.

There are still spaces for improvement. For example, the DET kernel only contains the structure of parse tree and neglects the dependency path tree. When the dependency tree kernel is combined with SPT tree kernel, the performance is expected to be improved [7]. Yang et al. [7] once used a weighted linear combination of individual kernels instead of assigning the same weight to each individual kernel, which will be studied in our future work. Apart from this, how to improve the semantic kernel would be the core of future research, specifically, how to select more proper contexts, how to compute the semantic similarity more effectively and utilize more ontologies.
